# Cannabinol modulates the endocannabinoid system and shows TRPV1‐mediated anti‐inflammatory properties in human keratinocytes

**DOI:** 10.1002/biof.2122

**Published:** 2024-09-14

**Authors:** Camilla Di Meo, Daniel Tortolani, Sara Standoli, Francesca Ciaramellano, Beatrice Clotilde Angelucci, Annamaria Tisi, Salam Kadhim, Eric Hsu, Cinzia Rapino, Mauro Maccarrone

**Affiliations:** ^1^ Department of Veterinary Medicine University of Teramo Teramo Italy; ^2^ Department of Biotechnological and Applied Clinical Sciences University of L'Aquila L'Aquila Italy; ^3^ European Center for Brain Research (CERC) Santa Lucia Foundation IRCCS Rome Italy; ^4^ InMed Pharmaceuticals Inc. Vancouver BC Canada

**Keywords:** cannabinol, cytokines, endocannabinoid system, keratinocytes, skin inflammation

## Abstract

Cannabinol (CBN) is a secondary metabolite of cannabis whose beneficial activity on inflammatory diseases of human skin has attracted increasing attention. Here, we sought to investigate the possible modulation by CBN of the major elements of the endocannabinoid system (ECS), in both normal and lipopolysaccharide‐inflamed human keratinocytes (HaCaT cells). CBN was found to increase the expression of cannabinoid receptor 1 (CB_1_) at gene level and that of vanilloid receptor 1 (TRPV1) at protein level, as well as their functional activity. In addition, CBN modulated the metabolism of anandamide (AEA) and 2‐arachidonoylglicerol (2‐AG), by increasing the activities of *N*‐acyl phosphatidylethanolamines‐specific phospholipase D (NAPE‐PLD) and fatty acid amide hydrolase (FAAH)—the biosynthetic and degradative enzyme of AEA—and that of monoacylglycerol lipase (MAGL), the hydrolytic enzyme of 2‐AG. CBN also affected keratinocyte inflammation by reducing the release of pro‐inflammatory interleukin (IL)‐8, IL‐12, and IL‐31 and increasing the release of anti‐inflammatory IL‐10. Of note, the release of IL‐31 was mediated by TRPV1. Finally, the mitogen‐activated protein kinases (MAPK) signaling pathway was investigated in inflamed keratinocytes, demonstrating a specific modulation of glycogen synthase kinase 3β (GSK3β) upon treatment with CBN, in the presence or not of distinct ECS‐directed drugs. Overall, these results demonstrate that CBN modulates distinct ECS elements and exerts anti‐inflammatory effects—remarkably via TRPV1—in human keratinocytes, thus holding potential for both therapeutic and cosmetic purposes.

## Graphical abstract

The figure summarizes the ability of cannabinol to differently modulate gene/protein expression and functionality of endocannabinoid system (ECS) elements in the in vitro model of human keratinocytes (HaCaT cells). Cannabinol is also shown to exert anti‐inflammatory effects on the release of IL‐8, IL‐12, and IL‐31 (via TRPV1 receptor), as well as of IL‐10.

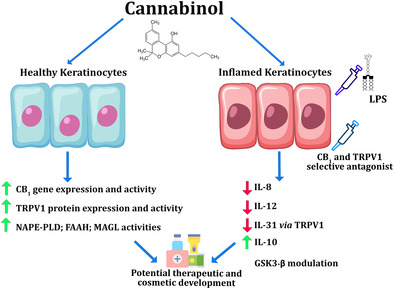

## 1 INTRODUCTION

Phytocannabinoids (pCBs) are a group of more than 150 different lipophilic compounds derived from cannabis (*Cannabis sativa*), a flowering plant native to Central Asia.^1^, ^2^ Cannabis has been cultivated for millennia with different aims such as nutrition, recreation, generation of seed oil and fibers for industrial purposes, as well as for religious and spiritual practices and, most interestingly, for medical applications.^3^ The best studied pCBs are Δ^9^‐tetrahydrocannabinol (THC) and cannabidiol (CBD),^2^, ^4^ although cannabis produces many other “minor” pCBs whose biological profile remains poorly investigated.^5^, ^6^ Recently, improved extraction and (bio)synthetic techniques have led to amounts of less abundant pCBs that are enough to investigate their biological activity.^7^ Among them, cannabinol (CBN) has recently attracted particular attention,^7^, ^8^ as a non‐psychoactive compound whose levels in cannabis flowers increase over time via the first conversion of THC acid (THCA) into THC, and the subsequent oxidation of THC into CBN.^8^, ^9^ Recent studies have suggested that CBN may have anti‐inflammatory, analgesic and antibacterial properties,^6^, ^7^, ^10^ although the mechanisms of action have still to be clarified.

In general, pCBs can interact with the endocannabinoid system (ECS),^7^, ^9^, ^11^ a complex network composed of endocannabinoids (eCBs), their receptors, metabolic enzymes and transporters, which altogether regulate various physiological processes and maintain homeostasis in the human body,^12^, ^13^ skin included.^14^, ^15^ Anandamide (*N*‐arachidonoylethanolamine, AEA) and 2‐arachidonoylglycerol (2‐AG) are the best studied eCBs, that are both synthesized from membrane phospholipid precursors via different biosynthetic pathways.^12^, ^16^, ^17^, ^18^, ^19^, ^20^ In particular, the cleavage of *N*‐arachidonoyl phosphatidylethanolamine (NArPE) by *N*‐acyl phosphatidylethanolamines‐specific phospholipase D (NAPE‐PLD) produces AEA,^21^, ^22^ that is hydrolyzed by fatty acid amide hydrolase (FAAH).^23^ 2‐AG is generated from the cleavage of diacylglycerol (DAG) by the two isoforms of diacylglycerol lipase (DAGL) α and β,^24^ which exhibit distinct spatial and cellular distributions. DAGLα is predominantly localized in the brain,^25^, ^26^ while DAGLβ is primarily expressed in immune cells including microglia, macrophages, and dendritic cells, being also involved in inflammatory processes.^27^, ^28^, ^29^ Then, 2‐AG is cleaved by monoacylglycerol lipase (MAGL).^23^, ^30^ The two main receptors responsible for the biological activity of eCBs are the cannabinoid receptors 1 (CB_1_) and 2 (CB_2_), though other orphan G‐coupled receptors like the G protein‐coupled receptor 55 (GPR55), the transient receptor potential (TRP) vanilloid receptor 1 (TRPV1), and peroxisome proliferator‐activated nuclear receptors (PPARs) α, γ, δ can be activated by eCBs.^11^ Remarkably, the main components of the ECS have been found in human keratinocytes, which are the predominant cells of the epidermis.^15^, ^31^, ^32^, ^33^, ^34^ ECS activation in keratinocytes has been shown to impact on various processes engaged in the regulation of skin physiology, while the dysregulation of the same system is linked to different skin diseases.^14^, ^15^, ^31^ In this context, we have recently shown that minor pCBs [i.e., cannabigerol (CBG), cannabichromene (CBC), Δ^9^‐tetrahydrocannabivarin (THCV) and cannabigerolic acid (CBGA)] modulate the expression and functional activity of distinct ECS elements in HaCaT cells,^35^ and block skin inflammation.^36^ Here, we sought to extend these observations to CBN, by using lipopolysaccharide (LPS)‐inflamed HaCaT cells. Taken together, our data reveal different CBN‐induced modulations of the ECS, as well as its anti‐inflammatory effect by reducing IL‐31 release through a TRPV1‐dependent mechanism.

## 2 MATERIALS AND METHODS

### 2.1 Materials

Dulbecco's modified Eagle medium‐high glucose (DMEM‐HG) and fetal bovine serum (FBS) were purchased from Corning Incorporated (NY, USA); antibiotics (penicillin/streptomycin, pen/strep), Dulbecco's phosphate buffer saline (D‐PBS without calcium and magnesium), and trypsin (2.5%) were from Gibco by Life Technologies (Thermo Fisher Scientific Company, Waltham, MA, USA); ethylenediamine tetraacetic acid (EDTA, 0.5 M) was from Invitrogen (Thermo Fisher Scientific Company, Waltham, MA, USA); Opti‐MEM was from Thermo Fisher Scientific (Waltham, MA, USA). CBN was purchased from Cerilliant Corporation (Sigma Aldrich Company, St. Louis, MO, USA). LPS from *Escherichia coli* O111:B4 (suitable for cell culture) and hydrocortisone (HC) were purchased from Sigma Aldrich (St. Louis, MO, USA). The agonist capsaicin, the antagonists SR141716A and CPZ were obtained from Sigma Aldrich (St. Louis, MO, USA). 3‐(4,5‐Dimethylthiazol‐2‐yl)‐2,5‐diphenyltetrazolium bromide (MTT) reagent was purchased from Sigma‐Aldrich (St. Louis, MO, USA) and dimethyl sulfoxide (DMSO) was from PanReac AppliChem (Glenview, IL, USA). ELISA kit to detect apoptosis was purchased from Roche Diagnostic (Basilea, Switzerland), while ELISA selective kits for each interleukin were purchased from R&D System DuoSet (Bio‐Techne, Minneapolis, MN, USA). Supplies for the RT‐qPCR, including the RevertAid H Minus First Strand cDNA Synthesis Kit and SensiFAST SYBR Lo‐ROX Kit, were obtained from Thermo Fisher Scientific (Waltham, MA, USA) and Bioline (Meridian Bioscience Inc. Company, Cincinnati, OH, USA), respectively, whereas supplies for Western blotting were purchased from Bio‐Rad Laboratories (Hercules, California, USA). [3H]CP55.940, CP55.940, and [3H]AEA were from PerkinElmer Life Sciences (Waltham, MA, USA); Fluo‐3 AM and Pluronic F‐127 were from Molecular Probes (Thermo Fisher Scientific, Waltham, MA, USA); N‐((6‐(2,4 dinitrophenyl)amino)hexanoyl)‐2(4,4‐difluoro‐5,7‐dimethyl‐4‐bora‐3a,4a‐diaza‐s indacene‐3‐pentanoyl)‐1‐hexadecanoyl‐sn‐glycero‐3 phosphoethanolamine, triethylammonium salt (PED6) was from Thermo Fisher Scientific (Waltham, MA, USA); p‐nitrophenyl butyrate (pNPB) was from Sigma Aldrich (St. Louis, MO, USA) and KT172 and arachidonoyl‐1‐thio‐glycerol were from Cayman Chemical (Ann Arbor, MI, USA). Human/mouse MAPK phosphorylation array was obtained from RayBiotech (RayBiotech Inc., Peachtree Corners, GA, USA).

### 2.2 Cell line and treatments

Immortalized HaCaT cells of Caucasian skin type (phototype) from the original depositor (DKFZ, Heidelberg) were purchased from Cell Lines Service (CLS Cell Lines Service, 300493). In all the experiments, HaCaT cells were used at the 5th–6th passage after thawing, cultured at 37°C in a humidified 5% CO_2_ atmosphere in DMEM‐HG and supplemented with 10% FBS and 1% antibiotic–antimycotic (pen/strep) solution. For MTT and apoptosis assays, RT‐qPCR, western blotting, receptors binding, and enzymes activity assays, cells were seeded in specific plates and, after 24 h, the cell medium was replaced with DMEM‐HG supplemented with 1% FBS and 1% pen/strep (starvation medium) and incubated for 6, 12, 24, and/or 48 h in the presence of CBN at specific concentrations (μM) according to each analysis. For the ELISA assay and MAPK array, performed in inflammatory conditions, cells were starved with DMEM‐HG with 1% FBS and 1% pen/strep for 24 h and then incubated as the following conditions: control (DMEM‐HG + 5% FBS + 1% pen/strep); LPS at the concentration of 5.0 μg/mL, positive control HC at 10 μM; CBN at 2.5 μM alone and in combination with the selective CB_1_ antagonist (SR141716A) at 0.1 μM^37^ and the selective TRPV1 antagonist CPZ at 5.0 μM.^38^ After 24 and 48 h, according to specific analysis, cell pellets and supernatants (medium) were collected from each condition, and then centrifuged at 300*g* for 5 min and stored at −20 and at −80°C, respectively.

### 2.3 MTT cytotoxicity assay

HaCaT cells were seeded into 96‐well plates at a density of 1 × 10^4^ per well and the day after were treated with vehicle (0.8% methanol) and CBN at different concentrations (0.5, 1.0, 2.5, 5.0, 10, and 25 μM), for the timepoints of 6, 12, and 24 h. After each timepoint, cells were incubated with MTT reagent at the final concentration of 0.5 mg/mL for 4 h. Then, the MTT solution was discarded, and DMSO was added to dissolve the formazan crystals. Cell viability was assessed by the reduction of the MTT reagent to purple formazan, by measuring the optical densities (OD) values at the EnSpire Multimode Plate Reader (Perkin Elmer, Waltham, MA, USA). Cell viability was expressed as percentage of control (100%), calculated by subtracting the 630 nm OD background from the 570 nm OD total signal for each sample, followed by the subtraction of cell‐free blank wells, as reported.^35^

### 2.4 Determination of apoptosis

HaCaT cells were seeded into 96‐well plates at a density of 1 × 10^4^ per well and the day after were treated with CBN at different concentrations (0.5, 1.0, 2.5, 5.0, 10, and 25 μM) for 24 h. Apoptotic cell death was quantified by an ELISA assay (Cell Death Detection ELISAPLUS, #11774425001, Roche Diagnostic, Basilea, Switzerland), based on the evaluation of DNA fragmentation through an immunoassay for histone‐associated DNA fragments in the cell cytoplasm. The ELISA kit also provides a positive control, represented by a DNA–histone‐complex. Specific determination of mono‐ and oligonucleosomes in the cytoplasmic fraction of cell lysates was performed by measuring the absorbance values at 405 nm, as reported.^39^

### 2.5 Quantitative real‐time polymerase chain reaction

HaCaT cells were seeded into six‐well plates at a density of 4 × 10^5^ per well, incubated overnight, and then exposed to CBN at 2.5 μM for 24 h. After treatments, cell pellets were collected and stored at −20°C. Total RNA was extracted from the cell pellets by using QIAzol Lysis Reagent (Qiagen, Hilden, Germany), according to manufacturer's instructions, and then quantified with a NanoDrop™ 2000/2000c Spectrophotometer (Thermo Fisher Scientific Company, Waltham, MA, USA). Five hundred nanograms of isolated mRNA was reverse transcribed in a final volume of 20 μL, later diluted 1:3, following incubation in a thermocycler for 5 min at 65°C, 5 min at 25°C, 60 min at 42°C, and 5 min at 70°C, by using the RevertAid H Minus First Strand cDNA Synthesis (Thermo Fisher Scientific Company, Waltham, MA, USA). Quantitative real‐time polymerase chain reaction (RT‐qPCR) was performed in a final volume of 10 μL, consisting of 1 μL of cDNA, 1 μL of primers Forward + Reverse (10 μM mix) and 5 μL of Sybr Green 1× (SensiFAST SYBR Lo‐ROX Kit, Bioline by Meridian Bioscience Inc., Cincinnati, OH, USA), in the Applied Biosystems 7500 Fast Real‐Time PCR System (Life Technologies, Carlsbad, CA, USA). The qPCR reaction was performed with the following conditions: holding at 50°C for 2 min and 95°C for 10 min, cycling at 95°C for 15 s and 60°C for 30 s (for 40 cycles) and a dissociation curve (melting curve) in the range of 60–95°C,^40^ to evaluate the specificity of the amplification products. After assigning a fluorescence threshold above background (threshold cycle number or *C*_t_), differences in threshold cycle number were used to quantify the relative amount of each PCR target. The relative expression of different amplicons was calculated by the delta–delta *C*_t_ (ΔΔ*C*_t_) method and was converted to relative expression ratio (2−^ΔΔ*C*t^) for statistical analysis.^41^ All data were normalized to the endogenous reference genes β‐actin and glyceraldehyde‐3‐phosphate dehydrogenase (GAPDH). The specific primers used were designed with Primer3 and ordered from Integrated DNA Technologies (IDT; Coralville, IA, USA), and their specific sequences are indicated in Table S2.

### 2.6 Western blotting

HaCaT cells were seeded into six‐well plates at a density of 4 × 10^5^ per well, incubated overnight, and then treated with CBN at 2.5 μM for 24 h. Cell pellets were collected and then lyzed in ice‐cold RIPA lysis buffer (Thermo Fisher Scientific Company, Waltham, MA, USA) in combination with the protease inhibitor cocktail 100× (Sigma Aldrich, St. Louis, MO, USA). The amount of protein was determined by the Bio‐Rad Protein assay (Bio‐Rad Laboratories, Hemel Hempstead, UK). An equal amount of protein (60 μg) for each sample was loaded onto 10% sodium dodecyl sulfate (SDS)–polyacrylamide gels and blotted onto polyvinylidene fluoride (PVDF) sheets (Amersham Biosciences, Piscataway, NJ, USA). Membranes were blocked with 5% non‐fat dried milk for 1 h at room temperature, and then incubated with suitable primary antibodies. Detection was performed using Azure Biosystems c400 (Dublin, CA, USA). The primary antibodies used were obtained from various sources, as detailed in Table S3.

### 2.7 CB_1_ binding assay

HaCaT cells were seeded in 12‐well plates at a density of 2 × 10^5^ cells per well under the following conditions, each in triplicate: total binding (TB), non‐specific binding (NSB), CB_1_ receptor antagonist (SR141716), and blanks (Bk). Cells were then treated with 2.5 μM CBN for 24 h. After two washes with 1 mL PBS, each well was incubated with 500 μL of incubation buffer (50 mM Tris–HCl, 5 mM MgCl_2_, 1 mM CaCl_2_, 0.2% BSA, and pH 7.4) preheated to 37°C for 15 min. The incubation buffer contained either 1 μM non‐radiolabelled CP55.940 for NSB wells, or 0.1 μM SR141716 for SR1 wells.^37^, ^42^ Subsequently, 2.5 nM [^3^H]CP55.940 was added to all wells, and the plates were incubated for 1 h at 37°C. This step allowed the radiolabeled ligand to compete with the pre‐incubated ligands for binding sites on the CB_1_ receptors. Then, the buffer was removed and the cells were washed again with an ice‐cold washing buffer (50 mM Tris–HCl, 500 mM NaCl, 0.1% BSA, and pH 7.4) to remove unbound radioactive material. Finally, 0.5 M NaOH (500 μL) was added to each well to lyse the cells. Cell lysates were transferred to 10 mL scintillation vials containing liquid scintillation cocktail, and radioactivity was measured by using a scintillation β‐counter (Tri‐Carb 2810 TR, Perkin Elmer, Waltham, MA, USA) as previously described.^43^

### 2.8 TRPV1 receptor activity assay

HaCaT cells (1 × 10^6^ cells) in suspension were incubated with 4 μM of the selective intracellular fluorescent probe Fluo‐3 AM containing 0.02% Pluronic F‐127 in Opti‐MEM medium, for 15 min at 25°C. Then, cells were washed in Tyrode's buffer (145 mM NaCl; 2.5 mM KCl; 1.5 mMCaCl_2_; 1.2 mM MgCl; 10 mM D‐glucose, 10 mM HEPES, and pH 7.4), resuspended in 2 mL of Tyrode's buffer and transferred to the quartz cuvette of the LS50B spectrofluorometer (Perkin Elmer, Waltham, MA, USA). Fluorescence was measured at 25°C (excitation at *λ* = 488 nm; emission at *λ* = 516 nm) from HaCaT cells pre‐incubated with CBN at 2.5 μM, and then stimulated with the selective TRPV1 agonist capsaicin (1 μM), as reported.^44^ TRPV1 activity was expressed as fluorescence intensity (arbitrary units, AU) per 10^6^ cells, as reported.^35^

### 2.9 Enzyme assays

#### 2.9.1 NAPE‐PLD assay

HaCaT cells were seeded into six‐well plates at a density of 4 × 10^5^ per well, incubated overnight, and then treated with CBN at 2.5 μM for 24 h. Cell pellets were collected and lyzed in a reaction mixture containing 50 mM Tris–HCl, 0.05% Triton X‐100 pH 8.0, and 10 μM of fluorogenic substrate PED6. Cell homogenates were pre‐incubated with the specific NAPE‐PLD inhibitor ARN19874 at 3.8 mM^45^ for 30 min at room temperature, to fully erase enzyme activity as a control. Fluorescence values were measured with excitation at *λ* = 485–488 nm, and emission at *λ* = 530 nm at 37°C, by means of continuity kinetics with 30‐s intervals for 30 min in an Enspire multimode plate reader (Perkin Elmer, Waltham, MA, USA), as reported.^46^ NAPE‐PLD activity was expressed as fluorescence intensity (arbitrary units, AU) per min per mg of protein, as reported.^35^

#### 2.9.2 FAAH assay

HaCaT cells were seeded into six‐well plates at a density of 4 × 10^5^ per well, incubated overnight, and then treated with CBN at 2.5 μM for 24 h. Cell pellets were collected and FAAH activity was determined in membranes isolated from these cells. Briefly, membranes were incubated with 10 μM [^3^H]AEA and 50 mM Tris–HCl, 0.05% BSA at pH 7.4 at 37°C in water bath for 30 min. The reaction was blocked with 1 mL chloroform/methanol (1:1; v/v) and centrifuged to 900*g* for 10 min at 4°C. The aqueous (top) layer was collected and transferred into scintillation vials. The amount of [3H]ethanolamine released was expressed as pmol per min per mg of protein, as reported.^47^

#### 2.9.3 DAGLα/β assay

HaCaT cells were seeded into six‐well plates at a density of 4 × 10^5^ per well, incubated overnight, and then treated with CBN at 2.5 μM for 24 h. Cell pellets were collected and DAGLα/β activity was determined in membranes isolated from these cells, by using the synthetic substrate pNPB (8 mM) in 50 mM HEPES pH 7.3, at room temperature for 20 min.^48^ The DAGLα/β inhibitor KT172 (1 μM) was used to full erase enzyme activity as a control, as reported.^48^

#### 2.9.4 MAGL assay

HaCaT cells were seeded into six‐well plates at a density of 4 × 10^5^ per well, incubated overnight, and then treated with CBN at 2.5 μM for 24 h. Cell pellets were collected and MAGL activity was determined in membranes isolated from these cells, by using the synthetic substrate arachidonoyl‐1‐thio‐glycerol (200 μM) in 10 mM Tris/1 mM EDTA pH 7.2, at 4°C for 15 min, and the MAGL inhibitor JZL184 (4 μM), to full erase enzyme activity, as reported.^48^

### 2.10 Enzyme‐linked immunosorbent assay

HaCaT cells were seeded into 12‐well plates at a density of 2 × 10^5^ per well, incubated overnight and then treated as follows: untreated (control) cells, 2.5 μM CBN, 5.0 μg/mL LPS, 10 μM HC, and 2.5 μM CBN. In addition, HaCaT cells were exposed to CBN in combination with the selective CB_1_ and TRPV1 antagonists (SR141716A at 0.1 μM and CPZ at 5.0 μM, respectively) for 24 h in the case of IL‐31 and for 48 h in the case of IL‐12. After each treatment, supernatants (medium) of each condition were collected, centrifuged at 4°C at 300*g* for 5 min and stored at −80°C until the assay. Cytokine quantification was performed using the DuoSet ELISA kit (#DY201, #DY208, #DY217B, #DY1270, and #DY2824, R&D Systems, Minneapolis, MN, USA), following the manufacturer's instructions. Briefly, 96‐well plates were coated overnight with the specific capture antibody for each interleukin and then blocked with 1% BSA. Selective standard proteins in duplicate and samples in triplicate were added and incubated for 2 h. The standard curve for each interleukin was generated by using serially diluted standard proteins at known concentrations as provided by the manufacturer, and the calibration curves obtained were used to interpolate sample values. Each well with standards and/or samples was incubated with the specific detection antibody for 2 h. The streptavidin–HRP was added and incubated for 20 min, followed by the substrate solution for another 20 min, and then the reaction was stopped with the specific Stop Solution (1 M H_2_SO_4_). Optical densities were determined using an Enspire microplate reader (Perkin Elmer, Waltham, MA, USA) at the specific wavelengths of 450 and 570 nm, as reported.^36^

### 2.11 MAPK signaling pathway array

HaCaT cells were seeded into 100 mm plates at a density of 8 × 10^5^ per well, incubated overnight and then exposed to 24 h treatment with LPS at 5.0 μg/mL, positive control HC at 10 μM, CBN at 2.5 μM both alone and in combination with the selective CB_1_ and TRPV1 antagonists (SR141716A at 0.1 μM and CPZ at 5.0 μM, respectively). The signaling pathway was analysed through a C‐Series Human/Mouse MAPK phosphorylation array, according to manufacturer recommendations (#AAH‐MAPK‐1‐8, RayBiotech Inc., Peachtree Corners, GA, USA). Briefly, proteins were extracted from HaCaT cell pellets and quantified using the BCA method. 500 μg of proteins were added into each nitrocellulose membrane coated with the specific antibody and incubated overnight at 4°C. The day after, each well was incubated with the specific detection antibody and with streptavidin–HRP for 2 h at room temperature. Chemiluminescent readings were taken using a C‐DiGit Blot Scanner (LI‐COR Bioscience, Lincoln, NE, USA), and densitometry data were extracted using Image Studio™ software (LI‐COR Bioscience, Lincoln, NE, USA). Readings were normalized to the positive loading controls, and the membrane background signal was subtracted, as reported.^36^

### 2.12 Statistical analysis

Data were analysed using the GraphPad Prism 8 program (GraphPad Software, La Jolla, CA, USA) and were reported as means ± SEM of three independent experiments. Statistical analysis was performed using the unpaired *t*‐test, multiple *t*‐test, and the one‐way analysis of variance (ANOVA), according to the data set, followed by a Bonferroni post‐hoc test. A value of *p* < 0.05 was considered statistically significant.

## 3 RESULTS

### 3.1 CBN modulates distinct ECS elements at gene and protein levels

**FIGURE 1:**
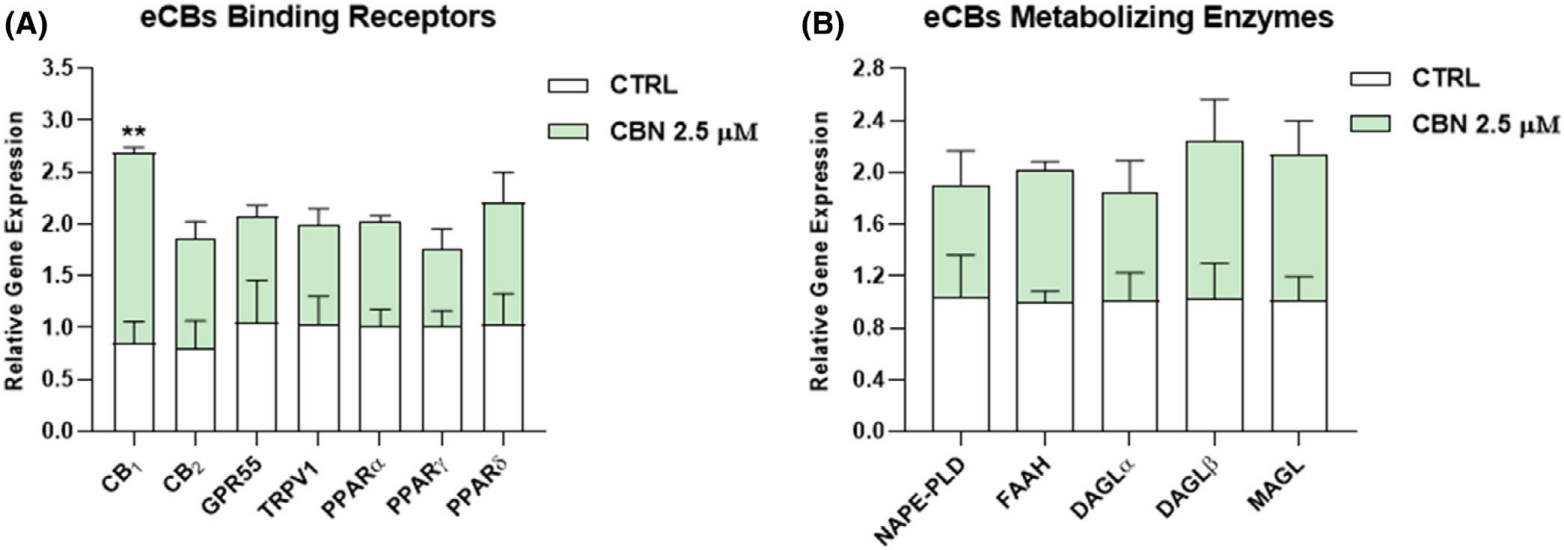
Gene expression of (A) eCB‐binding receptors (CB_1_, CB_2_, GPR55, TRPV1, and PPARα/γ/δ) and (B) eCBs metabolic enzymes (NAPE‐PLD, FAAH, DAGLα/β, and MAGL) in HaCaT cells following 24 h treatment with 2.5 μM CBN. The values were expressed as 2^(−ΔΔ*C*t)^ and normalized to β‐actin and GAPDH as housekeeping genes. Data are the mean ± SEM of three independent experiments (*n* = 3). Statistical analysis was performed using a multiple *t*‐test, followed by the correction with the Bonferroni–Dunn method (**p < 0.01 vs. control).

First, different concentrations of CBN—from 0.5 to 25 μM—were tested at three timepoints—6, 12, and 24 h—on HaCaT cells, through cell viability and apoptosis assays (Figure S1). A dose–response curve was plotted to determine the half‐maximal inhibitory concentration (IC_50_) of CBN at each timepoint, as indicated in Table S1. Based on these preliminary data, the non‐cytotoxic dose of 2.5 μM at 24 h (i.e., half of the calculated IC_50_) was selected to perform all subsequent analyses. Thus, the expression of ECS elements [eCB‐binding receptors (CB_1_, CB_2_, GPR55, TRPV1, and PPARα/γ/δ), and the main AEA (NAPE‐PLD, FAAH), and 2‐AG (DAGLα/β, MAGL) metabolic enzymes] was evaluated, at both mRNA—through quantitative real‐time polymerase chain reaction (RT‐qPCR)—and protein level—through Western blotting—in HaCaT cells after treatment with 2.5 μM CBN for 24 h. Notably, no alterations were found at gene level for any of the ECS elements tested, with the exception of CB_1_ which was significantly increased by CBN compared to control cells (*p* < 0.01 vs. CTRL) (Figure 1).

**FIGURE 2:**
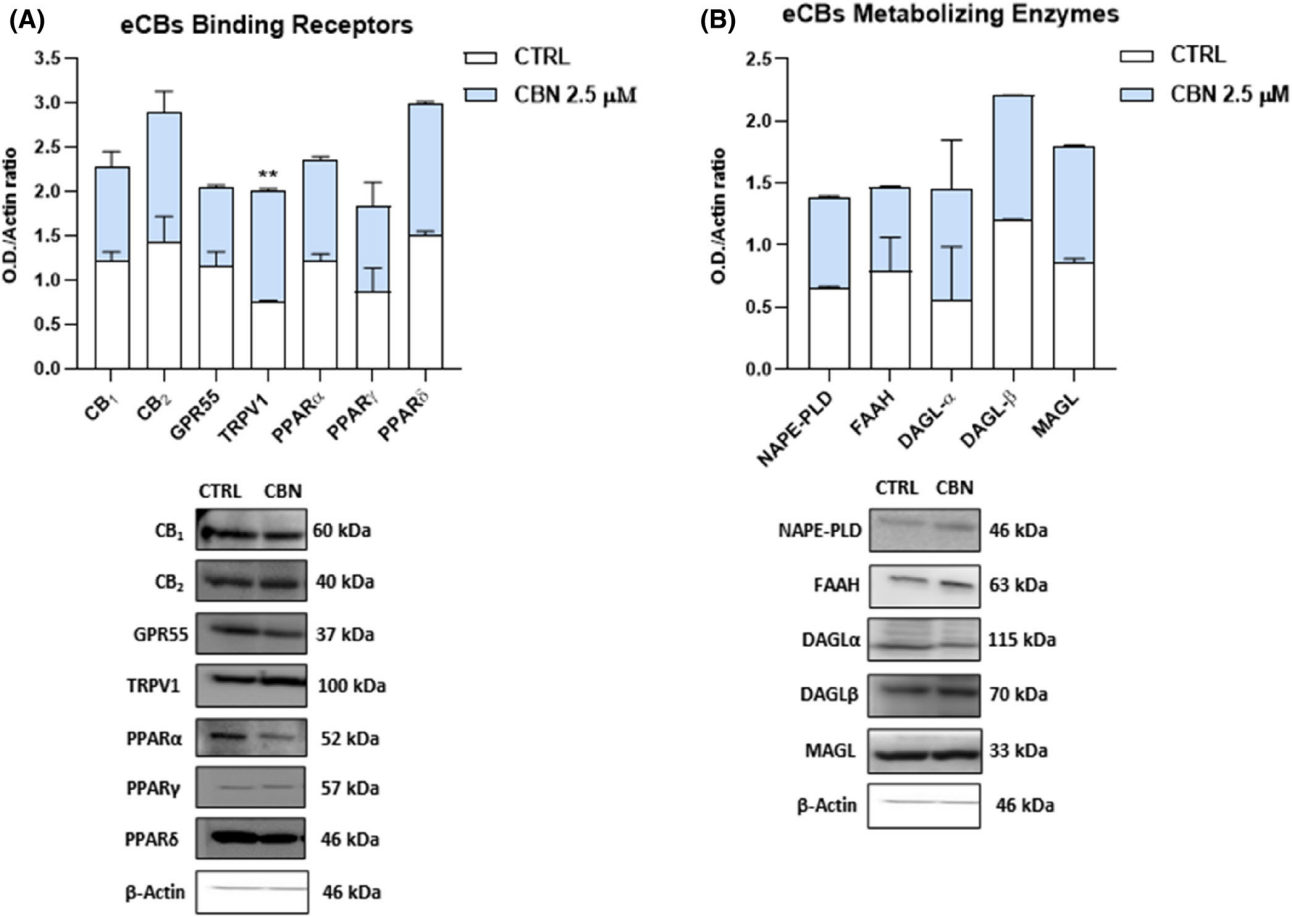
Protein expression of (A) eCBs‐binding receptors (CB_1_, CB_2_, GPR55, TRPV1, and PPARα/γ/δ) and (B) metabolic enzymes (NAPE‐PLD, FAAH, DAGLα/β, and MAGL) in HaCaT cells following 24 h treatment with 2.5 μM CBN. Densitometric analyses of immunoreactive bands were normalized to β‐actin as housekeeping protein. Data are the mean ± SEM of three independent experiments (*n* = 3). Statistical analysis was performed using a multiple *t*‐test, followed by the correction with the Bonferroni–Dunn method (**p < 0.01 vs. control). Representative bands of the main ECS components are shown in the lower panels.

As for the protein expression, only that of TRPV1 was significantly increased (*p* < 0.01 and *p* < 0.05, respectively vs. CTRL) in HaCaT cells exposed to 2.5 μM CBN, while the other ECS components remained unaffected under the same experimental conditions (Figure 2).

### 3.2 CBN modulates ECS at the functional level

**FIGURE 3:**
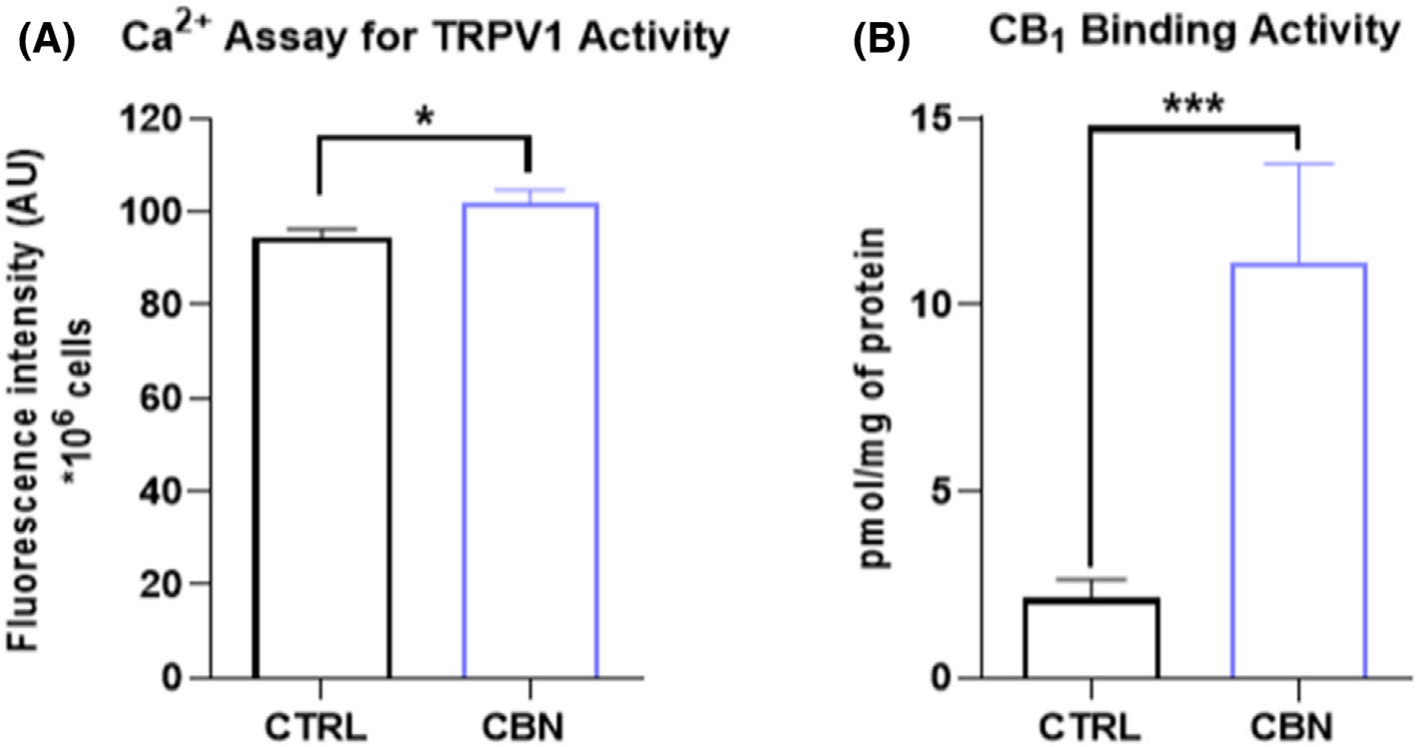
(A) Intracellular Ca^2+^ release triggered by capsaicin (a selective agonist of TRPV1) in HaCaT cells following 24 h treatment with 2.5 μM CBN. Data are means ± SEM of three independent experiments (*n* = 3). Statistical analysis was performed using an unpaired *t*‐test (*p < 0.05 vs. control). (B) CB_1_ binding activity in HaCaT cells following 24 h treatment with 2.5 μM CBN. Data are the mean ± SEM of three independent experiments (*n* = 3). Statistical analysis was performed using an unpaired *t*‐test (***p < 0.001 vs. control).

Then, the effect of CBN on functional activity of specific eCBs‐binding receptors and eCBs metabolic enzymes was investigated. In particular, the modulation of TRPV1 channel and CB_1_ receptor was assessed through specific intracellular Ca^2+^ release and radioligand binding assays, respectively. TRPV1 was activated by CBN in HaCaT cells, as shown by the significant increase in the calcium indicator (Fluo 3‐AM) fluorescence intensity in CBN‐treated cells (*p* < 0.05 vs. CTRL) (Figure 3A). Similarly, the CBN‐mediated activation of CB_1_ receptor was demonstrated by the significant increase (*p* < 0.001 vs. CTRL) in the binding activity, up to 3‐fold compared to control cells (Figure 3B).

**FIGURE 4:**
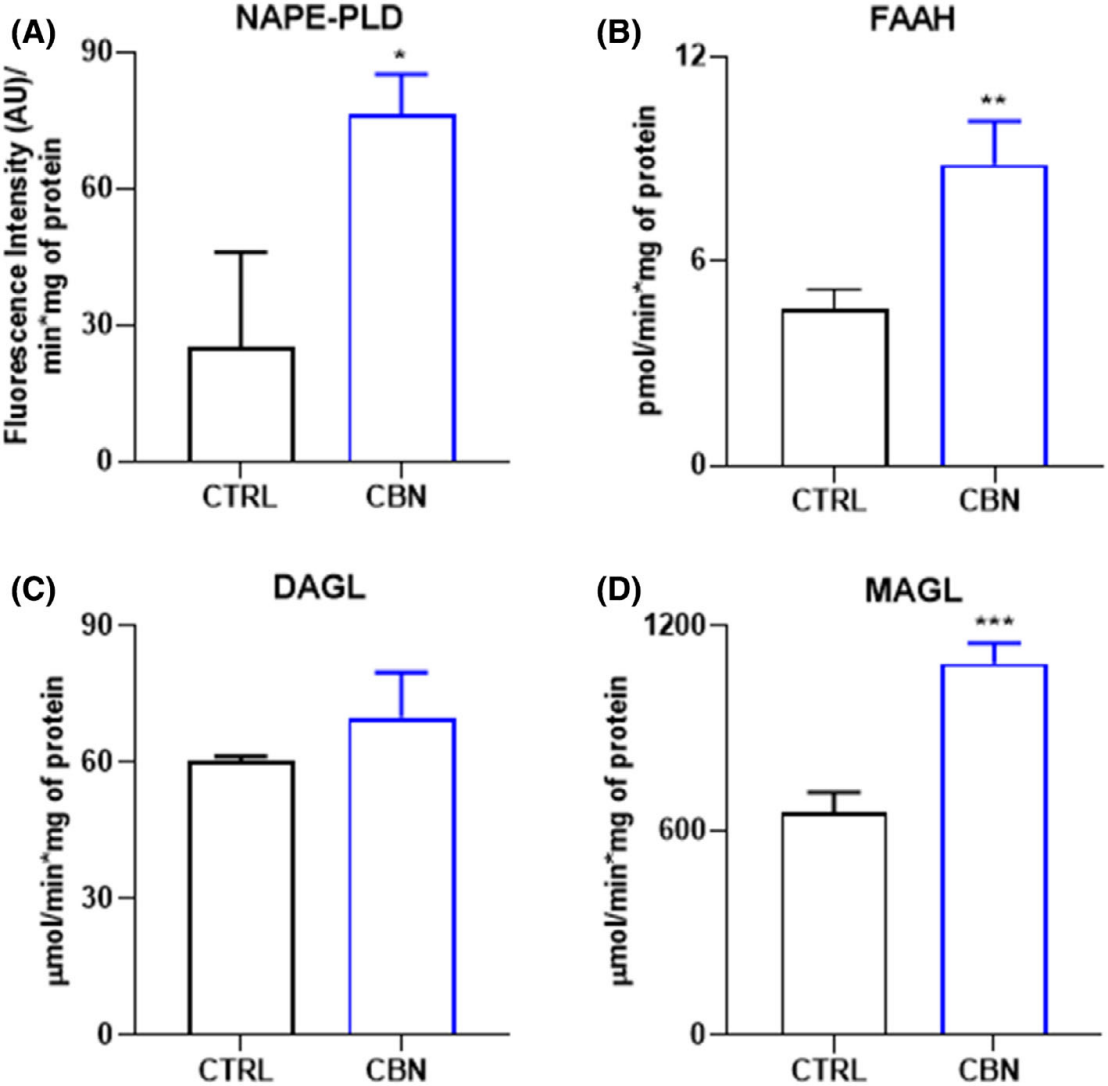
Activity of eCBs‐metabolic enzymes: (A) NAPE‐PLD, (B) FAAH, (C) DAGLα/β, and (D) MAGL, in HaCaT cells following 24 h treatment with 2.5 μM CBN. Data are the mean ± SEM of three independent experiments (*n* = 3). Statistical analysis was performed using an unpaired *t*‐test (*p < 0.05, **p < 0.01, and ***p < 0.001 vs. control).

The activity of AEA and 2‐AG metabolic enzymes was evaluated through specific enzymatic assays under the same experimental conditions. At 2.5 μM, CBN stimulated AEA metabolism since both NAPE‐PLD and FAAH activities increased in HaCaT cells compared to controls (*p* < 0.05 and *p* < 0.01, respectively vs. CTRL) (Figure 4A,B). As for 2‐AG metabolism, MAGL activity was significantly increased by CBN (*p* < 0.001 vs. CTRL), while no changes were observed for DAGLα/β activity (Figure 4C,D).

### 3.3 CBN prevents keratinocyte inflammation via TRPV1

**FIGURE 5:**
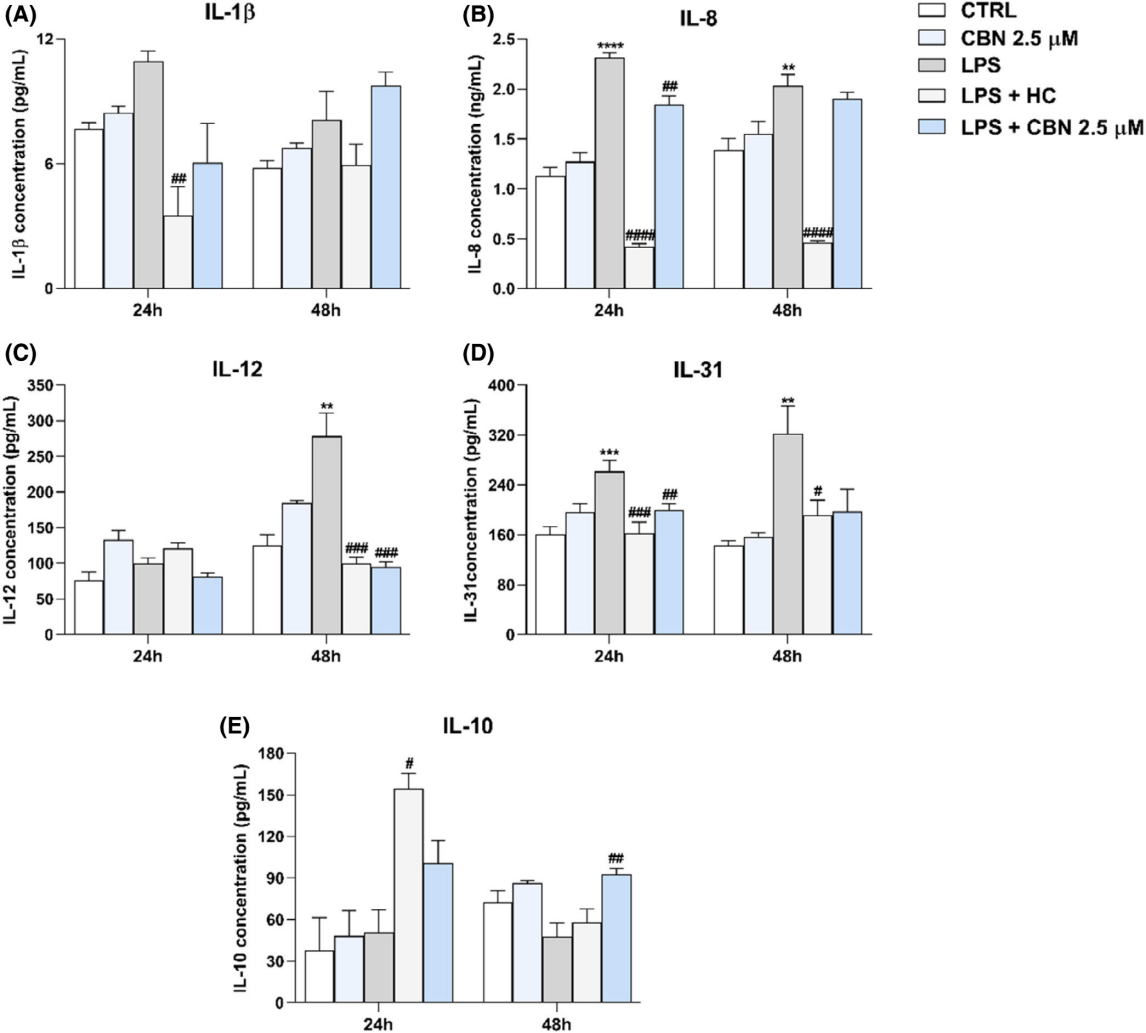
Release of ILs after HaCaT cells exposure to 2.5 μM CBN alone, 5.0 μg/mL LPS, 10 μM HC, and 2.5 μM CBN in the presence of LPS for 24 and 48h. (A) IL‐1β expression (pg/mL); (B) IL‐8 expression (ng/mL); (C) IL‐12 expression (pg/mL); (D) IL‐31 expression (pg/mL); and (E) IL‐10 expression (pg/mL). Data are presented as the mean ± SEM of three independent experiments (*n* = 3). Statistical analysis was performed using a one‐way ANOVA test followed by a Bonferroni post hoc test (**p < 0.01, ***p < 0.001, ****p < 0.0001 vs. CTRL; ^#^ *p* < 0.05, ^##^ *p* < 0.01, ^###^ *p* < 0.001, ^####^ *p* < 0.0001 vs. LPS).

To ascertain whether CBN may have anti‐inflammatory properties in human keratinocytes, as previously shown for other pCBs,^36^ the release of pro‐inflammatory ILs (IL‐1β, IL‐8, IL‐12, and IL‐31), and of the anti‐inflammatory IL‐10 was analysed through enzyme‐linked immunosorbent (ELISA) assay in inflamed HaCaT cells exposed to 5.0 μg/mL of LPS, with or without 2.5 μM CBN for 24 and 48 h, which represents the two timepoints previously used to set‐up the in vitro inflammatory model of keratinocytes.^36^ LPS treatment markedly increased the levels of all pro‐inflammatory cytokines, except for IL‐1β and for IL‐12 at 24 h. Notably, 2.5 μM CBN in combination with LPS significantly reverted the increase of IL‐8 (*p* < 0.01 vs. LPS) at 24 h of treatment, although to a lesser extent than the anti‐inflammatory compound hydrocortisone (HC), used as a positive control (Figure 5A,B). The anti‐inflammatory action of 2.5 μM CBN was more effective on IL‐12 and IL‐31 levels; indeed, 2.5 μM CBN significantly reverted the LPS‐mediated increase in IL‐12 (*p* < 0.001 vs. LPS) and IL‐31 (*p* < 0.01 vs. LPS) at 48 and 24 h, respectively, much alike HC (Figure 5C,D). In addition, IL‐31 levels were reduced by CBN at 48 h, yet in a non‐significant manner (Figure 5D). Moreover, the anti‐inflammatory IL‐10 was weakly modulated by LPS alone, whereas 2.5 μM CBN significantly increased its level (*p* < 0.01 vs. LPS) when cells were exposed to both LPS and CBN for 48 h (Figure 5E). Incidentally, treatment of HaCaT cells with CBN alone did not significantly affect the release of any of the tested cytokines (Figure 5).

**FIGURE 6:**
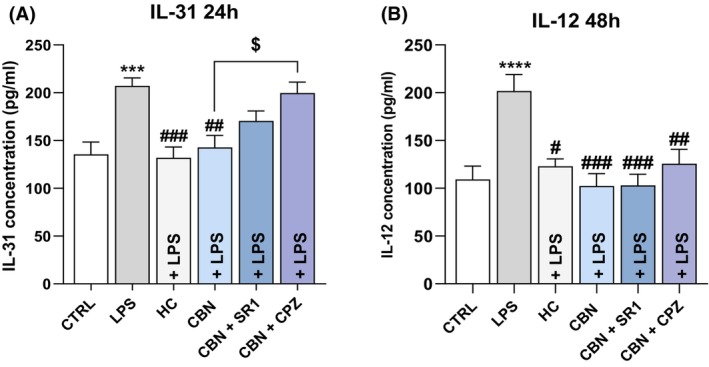
Release of ILs after HaCaT cells exposure to 5.0 μg/mL LPS, 10 μM HC, 2.5 μM CBN alone or in combination with the two selective antagonists for CB_1_ (SR1 at 0.1 μM) and TRPV1 (CPZ at 5.0 μM). (A) IL‐31 expression (pg/mL) after 24 h treatment and (B) IL‐12 expression (pg/mL) after 48 h treatment. Data are presented as the mean ± SEM of three independent experiments (*n* = 3). Statistical analysis was performed using a one‐way ANOVA test followed by a Bonferroni post hoc test (***p < 0.001, ****p < 0.0001 vs. CTRL; ^#^ *p* < 0.05, ^##^ *p* < 0.01, ^###^ *p* < 0.001 vs. LPS; ^$^ *p* < 0.05 vs. CBN).

To further investigate whether CBN may regulate IL‐12 and IL‐31 release in an ECS‐dependent manner, HaCaT cells were pretreated also with SR141716A (SR1)—selective antagonist of CB_1_—and capsazepine (CPZ)—selective TRPV1 antagonist—under inflammatory conditions (Figure 6A,B). Interestingly, CPZ significantly restored the anti‐inflammatory effect of CBN on IL‐31 release (*p* < 0.05 vs. CBN) in HaCaT cells exposed to LPS for 48 h, suggesting an engagement of TRPV1 (Figure 6A). Instead, the action of CBN on IL‐12 was not mediated by CB_1_ nor by TRPV1, as the reduction of IL‐12 caused by CBN was not affected by either SR1 or CPZ (Figure 6B).

### 3.4 Expression of mitogen‐activated protein kinases in inflamed keratinocytes

**FIGURE 7:**
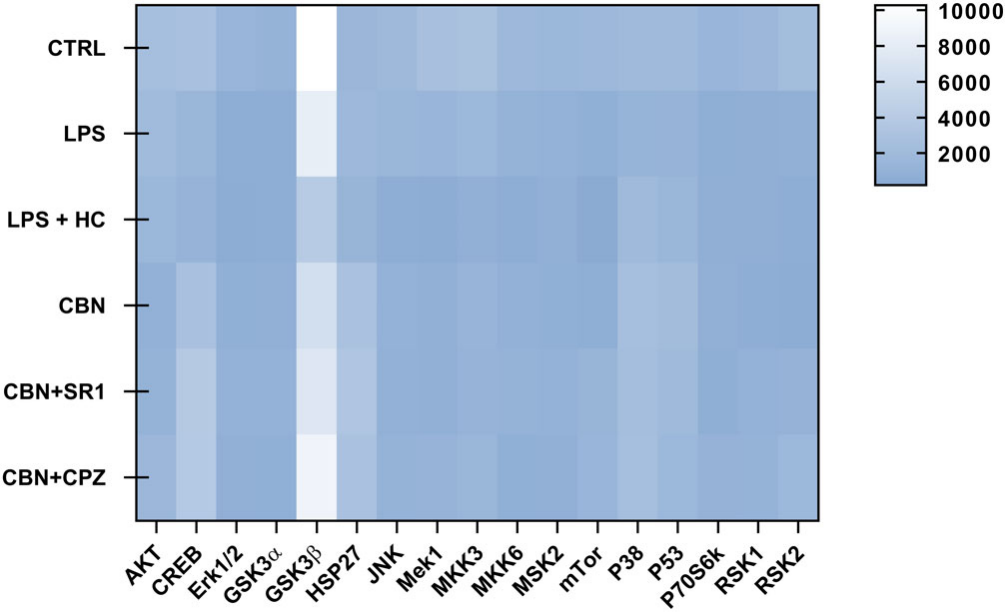
Heat map of the most relevant 17 proteins of the MAPK signaling pathway in HaCaT cells following 24 h treatment with LPS (5.0 μg/mL) in the presence of 2.5 μM CBN, alone or in combination with the two selective antagonists for CB_1_ (SR1 at 0.1 μM) and TRPV1 (CPZ at 5.0 μM). Samples were analyzed through the human phosphorylation array, and data are expressed as means of three sets of experiments for each condition (*n* = 3).

Since mitogen‐activated protein kinases (MAPK) pathway regulates the inflammatory responses of several cell types—keratinocytes included,^49^ the expression of 17 proteins of the MAPK signaling route was investigated in inflamed HaCaT cells exposed to CBN, alone or in combination with selective CB_1_ and TRPV1 antagonists, by using a human phosphorylation array (for details see Figure S2). The overall expression pattern of phosphorylated MAPK proteins was found to be low under most experimental conditions, apart from glycogen synthase kinase 3β (GSK3β) that was high in all experimental groups (Figure 7). Of note, GSK3β is the only MAPK analysed to be inactive in the phosphorylated form,^50^ and its expression was found to be modulated (yet not significantly) under all experimental conditions (Figure 7). As for the other MAPK proteins, only minor alterations in the expression of cyclic adenosine monophosphate response element‐binding protein (CREB), heat shock protein 27 (HSP27), p38 mitogen‐activated protein kinase (p38), and tumor protein p53 (p53) were observed in inflamed HaCaT cells treated with CBN in the presence of SR1 or CPZ, compared to LPS alone (Figure 7).

## 4 DISCUSSION AND CONCLUSION

To date, the effects of cannabis on skin physiology and pathology have focused on the main pCBs CBD and THC,^51^, ^52^, ^53^ while little is known about minor pCBs like CBN. Recently, we have shown that non‐psychotropic pCBs CBG, CBC, THCV, and CBGA differently modulate gene and protein expression of distinct ECS elements, as well as their functional activity in HaCaT cells.^35^ In line with this, here we demonstrate that in the same human keratinocytes CBN—used at a non‐cytotoxic half‐IC_50_ dose—increases gene and protein expression of specific eCBs‐biding receptors (i.e., CB_1_ and TRPV1, respectively), without modulating gene and protein expression of metabolic enzymes of these endogenous lipids. Of note, differences between gene and protein expression of ECS elements are not unprecedented.^32^, ^35^, ^54^, ^55^ Such discrepancies may be due to distinct half‐lives of mRNAs and proteins, which are caused by different degrading processes^56^ that lead to independent stability profiles.^57^, ^58^ At the functional level, CBN upregulates AEA metabolism, by stimulating the activity of NAPE‐PLD and FAAH, and promotes the activity of the degradative enzyme of 2‐AG, MAGL, but not that of its biosynthetic enzymes DAGLα/β. In addition, our functional data show that CB_1_ binding is significantly increased by CBN, a finding that is in keeping with previous literature data showing CBN activity on CB_1_ in monkey kidney (COS‐7) transfected cells, as well as in rat brain synaptosomal preparations.^59^ The engagement of TRPV1 by CBN was also investigated, because CBN was shown to be weakly effective on TRPV1, relatively potent on the transient receptor potential ankyrin 1 (TRPA1), and only weakly active on the TRP vanilloid receptor 2 (TRPV2) in HEK‐293 cells stably overexpressing recombinant rat or human TRP channels.^60^ Our present data extend previous findings to human keratinocytes, that constitutively express the TRPV1 channel, supporting the concept that CBN may have a promising pharmacological profile for skin‐related conditions by engaging this eCBs‐binding receptor channel. In line with this, we investigated the effects of CBN in an inflamed keratinocyte model consisting of HaCaT cells exposed to LPS.^36^ It is well known that the process of inflammation is orchestrated by inflammatory mediators, and a typical response evoked by pCBs is the dysregulation of cytokine production by immune cells with an unbalance between those produced by T‐helper subsets Th1 and Th2.^61^, ^62^ Here, we chose to evaluate the effect of CBN in our inflamed keratinocytes model on the release of some of the main cytokines known to be involved in the pathogenesis of skin diseases.^63^ Consistently, we found a reduction by CBN in the release of some pro‐inflammatory ILs at different time points of HaCaT stimulation (i.e., IL‐8 and IL‐31 at 24 h and IL‐12 at 48 h), while an increase of anti‐inflammatory IL‐10 release at 48 h. Among these, IL‐12 is a key cytokine involved in the regulation of epidermal functions,^64^ with possible inflammatory^65^ and protective^66^, ^67^ roles in the skin. The increase in the IL‐12p70 release in psoriasis and during the chronic phase of atopic dermatitis is thought to be responsible for controling the immune and inflammatory response, by promoting the differentiation of Th0 lymphocytes into Th1‐type cells.^67^, ^68^, ^69^ On the other hand, recent studies suggested a protective effect of IL‐12 in a psoriatic mouse model, due to the activation of a transcriptional programme that limits skin inflammation by engaging IL‐12 receptor signaling.^66^ Our data showed an increase in IL‐12 levels under inflammatory conditions, thus corroborating its role in inflammation, that was reverted by CBN. Moreover, IL‐31 is one of the most relevant cytokines in the context of the pathogenesis of pruritus.^70^, ^71^ Indeed, IL‐31 expression was found to be increased in lesional and non‐lesional skin of patients with atopic dermatitis,^72^, ^73^, ^74^ and it is able to induce severe pruritus in mice where levels of IL‐31 correlated with scratching behaviour.^75^ In addition to its pruritogenic function, IL‐31 also directly inhibits keratinocyte differentiation by downregulating the expression of proteins involved in barrier and differentiation processes, overall leading to the disruption of the epidermal barrier function.^76^, ^77^, ^78^ In the light of the preminent role of IL‐31 in skin inflammation, the ability of CBN to decrease its secretion appears noteworthy. More in general, the ability of CBN to decrease or increase the pro‐ and anti‐inflammatory outputs has been previously reported in different paradigms, for example in a murine model of ovalbumin‐sensitized allergic asthma where CBN blocked the production of ILs 2, 4, 5, 13 and decreased allergen mucus production.^10^ Similarly, CBN was shown to inhibit pro‐inflammatory cytokine production from oral bacteria‐exposed human monocytes and epithelial cells.^79^ More recently, CBN was also found to exert anti‐inflammatory effects by influencing different stages of gene expression: transcription, post‐transcriptional regulation, translation, and post‐translational regulation in human macrophages.^80^ It should be recalled that pCBs and pCB‐like compounds have proven potent anti‐inflammatory activity through mechanisms that are not yet fully understood. Under some circumstances, activation of CB_1/2_ receptors provides anti‐inflammatory responses, and particularly CB_2_ appears to be the main player in allergic contact dermatitis^81^, ^82^ and immune suppression of oral pathogens, where a CB_2_/phosphoinositide 3‐kinase (PI3K) axis is activated by CBN (as well as by CBD and THC).^79^ Conversely, CB_1/2_ receptors may not even be engaged in these processes, as demonstrated by a study where the topical application of THC effectively improved allergic contact dermatitis in both wild‐type and CB_1_/CB_2_‐deficient mice.^51^ In order to fill this gap knowledge, we decided to focus the attention on IL‐12 and IL‐31 to interrogate a possible correlation with the ECS. In this context, we previously observed beneficial effects induced by other minor pCBs (e.g., cannabigerol, cannabichromene, Δ^9^‐tetrahydrocannabivarin, and cannabigerolic acid) on IL‐31 via ECS involvement.^36^ Here, we obtained similar results with CBN, which was able to revert the inflammatory secretion of IL‐31 through the TRPV1 channel, while CB_1_ did not appear to be involved. Instead, the effects of CBN on the release of IL‐12 were found to be ECS‐independent. The correlation between IL‐31 and TRPV1 channel—in the presence of CBN—seems to call for particular attention, since TRPV1 is known to have pruritic functions and to increase in atopic dermatitis‐like skin lesions, both in humans and in a mouse model of psoriasiform dermatitis.^83^, ^84^, ^85^ There is also literature evidence that attests beneficial effects of TRPV1‐targeting. Notably, the eCB‐like compound PEA was found to inhibit allergic contact dermatitis in mice, as well as cytokine expression in human HaCaT cells, through TRPV1‐associated mechanisms.^86^ In addition, some pCBs stimulate TRPV1 channels, as reported by us and others.^35^, ^60^, ^87^, ^88^ Overall, these results and the key role of IL‐31 in inflammatory skin diseases, support the potential of TRPV1‐targeting (e.g., by CBN) for therapeutic purposes, for instance to treat itch‐associated skin conditions.^89^ Interestingly, antibodies against IL‐31 have shown to ameliorate the scratching behaviour in a mouse model of atopic dermatitis,^90^ and patients affected by chronic spontaneous urticaria responded to a treatment able to reduce IL‐31 serum levels.^91^

In this study, we also sought to elucidate signaling events that could explain the CBN‐mediated suppression of inflammation by analysing the modulation of phosphorylated proteins of the MAPK pathway. The activation of this pathway by LPS involves a series of phosphorylation events leading to the induction of inflammatory mediators, cytokines included.^92^, ^93^ Among the different MAPKs investigated in our inflamed model, the phosphorylated and inactive form of GSK3β appeared to be the most expressed in HaCaT cells under CBN stimulation and all other experimental conditions. A regulatory role of GSK3β in the modulation of inflammation has been already suggested.^94^, ^95^ In particular, the knock‐down of GSK3β was found to significantly reduce LPS‐associated pro‐inflammatory cytokines and death of human monocytes. Interestingly, cannabinoids have been reported to suppress neuroinflammation in Alzheimer's disease with a downregulation of phospho‐GSK3β.^96^ In contrast, the anti‐inflammatory signal evoked by CBN (as well as by CBD and THC) to suppress oral pathogens does not appear to be executed via the GSK3β‐dependent cholinergic anti‐inflammatory pathway.^79^ In our study, other phosphorylated active proteins of the MAPK family appear to be involved in the CBN anti‐inflammatory response, yet to a lesser and non‐significant extent.

In summary, in this investigation CBN has been shown to be active on inflamed human keratinocytes via TRPV1 channels, thus supporting a potential therapeutic effect of this minor pCB in the treatment of inflammatory skin diseases. It should be recalled that CBN has been indeed suggested as a potential component of topical applications to be used against psoriasis, where it inhibits keratinocyte proliferation through as yet unclear CB_1/2_‐independent mechanisms.^97^ A recent study has also shown anti‐melanoma, anti‐melanogenic, and anti‐tyrosinase properties of CBN (and other pCBs), thus making them useful as novel cosmeceutical products for skin care.^98^ Moreover, a CBN‐based therapy has been recently shown to be effective in the treatment of epidermolysis bullosa (EB), a group of rare medical conditions characterized by easy blistering of the skin and mucous membranes.^99^ This therapy was recently completed in a phase 2 clinical trial, aimed at assessing its potential as a topical treatment for EB, where it may help to relieve hallmark symptoms such as itch in a subset of EB Simplex patients.^99^ On a final note, it can be recalled that the therapeutic potential of CBN goes beyond the skin, because another CBN‐based topical eye drop formulation has been demonstrated to be effective in the treatment of glaucoma, where it may prevent the inflammation leading to elevated intraocular pressure and hence damage to the retinal ganglion cells.^100^

Taken together, our data suggest that CBN may hold true therapeutic potential to treat different human skin diseases. Such a biological activity of CBN occurs through engagement of selected elements of the endocannabinoid system—in particular TRPV1—a finding that paves the way to the development of distinct formulations of cannabis extracts for selected therapeutic applications.

## AUTHOR CONTRIBUTIONS

C. R. and M. M. contributed to conceptualization, writing‐reviewing and editing. C. D. M. and D. T. worked on validation, draft preparation and visualization. C. D. M, D. T., S. S., F. C., and C. B. A. worked on methodology and investigation. C.D.M, D.T., S.S., F.C, C.B.A. and A.T. worked on formal analysis. C. D. M., D. T., E. H., and S. K. dealt with resources. M. M. was in charge of supervision, project administration, and funding acquisition. All authors reviewed and approved the final version of the manuscript.

## FUNDING INFORMATION

This work was supported by InMed Pharmaceuticals Inc. (Vancouver, Canada) under a collaborative research agreement (No. PCI0029) with Professor Mauro Maccarrone. The funder had no role in the design of the study, in the collection, analyses or interpretation of data, nor in the decision to publish the results.

## CONFLICT OF INTEREST STATEMENT

The authors declare that the InMed Pharmaceuticals is commercializing cannabinoid‐based therapies. Eric Hsu is the Senior Vice President of this company; Salam Kadhim is a former Senior Scientist of Preclinical R&D, and Mauro Maccarrone is a Scientific Advisory Board member.

## Supporting information


**FIGURE S1:** Cell viability and apoptosis analysis. (A) Viability of HaCaT cells treated with vehicle (CTRL) or increasing concentrations (0.5, 1.0, 2.5, 5.0, 10, and 25 μM) of CBN at different timepoints (6, 12, and 24 h). Values are plotted as a logarithmic dose–response curve used to determine the IC_50_ (μM) for CBN at each time point. Data are means ± SEM of three independent experiments (*n* = 3). (B) Apoptosis of HaCaT cells treated with vehicle (CTRL) or increasing concentrations (0.5, 1.0, 2.5, 5.0, 10, and 25 μM) of CBN for 24 h. Data are means ± SEM of three independent experiments (*n* = 3). Statistical analysis was performed by one‐way ANOVA test followed by Bonferroni *post hoc* test (****p < 0.001 vs. CTRL).
**FIGURE S2:** MAPK kinases expression in the inflamed model of keratinocytes. (A) Representative images of phospho‐kinase arrays for each treatment, captured by C‐DiGit blot scanner. Each membrane detects the following 17 MAPKs: serine/threonine kinase 1 (AKT); cyclic adenosine monophosphate (cAMP) response element‐binding protein (CREB); glycogen synthase kinase 3 α (GSK3α) and ‐β (GSK3β); c‐Jun N‐terminal kinase (JNK); extracellular signal‐regulated kinase (ERK1); mitogen‐activated protein kinase (MEK1); mitogen‐activated protein kinase kinase 3 (MKK3) and 6 (MKK6); mitogen‐ and stress‐activated protein kinase 2 (MSK2); heat shock protein 27 (HSP27); mammalian target of rapamycin (mTor); p38 mitogen‐activated protein kinase (p38); tumor protein p53 (p53); p70 ribosomal S6 kinase (P70S6k); and ribosomal S6 kinase 1 (RSK1) and 2 (RSK2) (each spotted in duplicate). The pairs of dots in the upper corner on the left side are positive controls. (B) Distribution of antibodies for each of the 17 MAPK phosphorylated proteins on the membrane supplied by the array.
**TABLE S1:** IC_50_ values (μM) of CBN tested at three different time points (6, 12, and 24 h).
**TABLE S2:** Primers used for RT‐qPCR analyses. All the primers for the ECS and housekeeping genes were designed with Primer3 and ordered from Integrated DNA Technologies (IDT; Coralville, IA, USA).
**TABLE S3:** Primary antibodies used for western blotting analyses. All the primary antibodies used for ECS and for the housekeeping protein (β‐Actin) are indicated.
