# Supplementary material for: Cannabinol modulates the endocannabinoid system and shows TRPV1‐mediated anti‐inflammatory properties in human keratinocytes
Source: Biofactors. 2024 Sep 14;51(1):e2122. doi: 10.1002/biof.2122 (PMC11681214; doi:10.1002/biof.2122)
Supplement: Supplementary file 1 — FIGURE S1: Cell viability and apoptosis analysis. (A) Viability of HaCaT cells treated with vehicle (CTRL) or increasing concentrations (0.5, 1.0, 2.5, 5.0, 10, and 25 μM) of CBN at different timepoints (6, 12, and 24 h). Values are plotted as a logarithmic dose–response curve used to determine the IC50 (μM) for CBN at each time point. Data are means ± SEM of three independent experiments (n = 3). (B) Apoptosis of HaCaT cells treated with vehicle (CTRL) or increasing concentrations (0.5, 1.0, 2.5, 5.0, 10, and 25 μM) of CBN for 24 h. Data are means ± SEM of three independent experiments (n = 3). Statistical analysis was performed by one‐way ANOVA test followed by Bonferroni post hoc test (****p < 0.001 vs. CTRL). FIGURE S2: MAPK kinases expression in the inflamed model of keratinocytes. (A) Representative images of phospho‐kinase arrays for each treatment, captured by C‐DiGit blot scanner. Each membrane detects the following 17 MAPKs: serine/threonine kinase 1 (AKT); cyclic adenosine monophosphate (cAMP) response element‐binding protein (CREB); glycogen synthase kinase 3 α (GSK3α) and ‐β (GSK3β); c‐Jun N‐terminal kinase (JNK); extracellular signal‐regulated kinase (ERK1); mitogen‐activated protein kinase (MEK1); mitogen‐activated protein kinase kinase 3 (MKK3) and 6 (MKK6); mitogen‐ and stress‐activated protein kinase 2 (MSK2); heat shock protein 27 (HSP27); mammalian target of rapamycin (mTor); p38 mitogen‐activated protein kinase (p38); tumor protein p53 (p53); p70 ribosomal S6 kinase (P70S6k); and ribosomal S6 kinase 1 (RSK1) and 2 (RSK2) (each spotted in duplicate). The pairs of dots in the upper corner on the left side are positive controls. (B) Distribution of antibodies for each of the 17 MAPK phosphorylated proteins on the membrane supplied by the array. TABLE S1: IC50 values (μM) of CBN tested at three different time points (6, 12, and 24 h). TABLE S2: Primers used for RT‐qPCR analyses. All the primers for the ECS and housekeeping genes were designed with P [file BIOF-51-0-s001.docx]

**SUPPLEMENTARY MATERIALS**

**Cannabinol modulates the endocannabinoid system and shows TRPV1-mediated anti-inflammatory properties in human keratinocytes**

Camilla Di Meo^1,2#^, Daniel Tortolani^1#^, Sara Standoli^1^, Francesca Ciaramellano^3^, Beatrice Clotilde Angelucci^1^, Salam Kadhim^4^, Eric Hsu^4^, Cinzia Rapino^1^*, Mauro Maccarrone^2,3^*

^1^Department of Veterinary Medicine, University of Teramo, 64100 Teramo, Italy

^2^Department of Biotechnological and Applied Clinical Sciences, University of L'Aquila, 67100 L'Aquila, Italy

^3^European Center for Brain Research (CERC), Santa Lucia Foundation IRCCS, 00143 Rome, Italy

^4^InMed Pharmaceuticals Inc., Vancouver, BC V6C 1B4, Canada

^#^These authors contributed equally to this work

*Authors to whom correspondence should be addressed

***Analysis of cell viability and apoptosis***


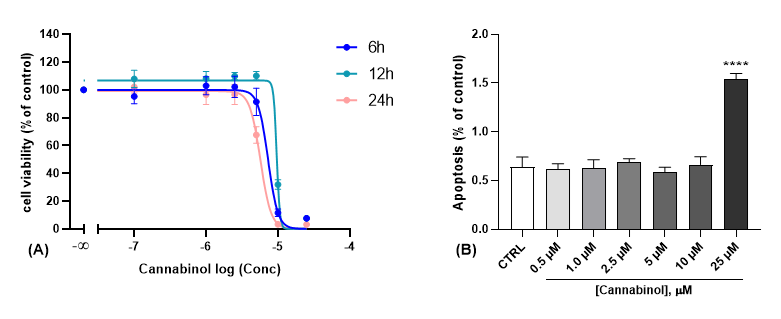


**Figure S1.** Cell viability and apoptosis analysis. **(A)** Viability of HaCaT cells treated with vehicle (CTRL) or increasing concentrations (0.5 µM, 1.0 µM, 2.5 µM, 5.0 µM, 10 µM, 25 µM) of CBN at different timepoints (6h, 12h and 24h). Values are plotted as a logarithmic dose-response curve used to determine the IC_50_ (µM) for CBN at each time point. Data are means ± SEM of three independent experiments (n=3). **(B)** Apoptosis of HaCaT cells treated with vehicle (CTRL) or increasing concentrations (0.5, 1.0, 2.5, 5.0, 10, 25 µM) of CBN for 24h. Data are means ± SEM of three independent experiments (n=3). Statistical analysis was performed by One-Way ANOVA test followed by Bonferroni *post hoc* test (****p<0.001 *vs* CTRL).

***Half-maximal inhibitory concentration (IC_50_) of CBN***

| **pCB** | **Time points (h)** | **IC_50_ (µM)** |
| --- | --- | --- |
| **CBN** | **6** | **6.9** |
|  | **12** | **9.6** |
|  | **24** | **5.1** |

**Table S1.** IC_50_ values (µM) of CBN tested at three different time points (6h, 12h, 24h).

***Quantitative Real-Time Polymerase Chain Reaction (RT-qPCR)***

**Table S2.** Primers used for RT-qPCR analyses. All the primers for the ECS and housekeeping genes were designed with Primer3 and ordered from Integrated DNA Technologies (IDT; Coralville, IA, USA).

| Gene | Forward Primer Sequence (5'→3') | Reverse Primer Sequence (5'→3') |
| --- | --- | --- |
| *CNR1* | CCTTTTGCTGCCTAAATCCAC | CCACTGCTCAAACATCTGAC |
| *CNR2* | TCAACCCTGTCATCTATGCTC | AGTCAGTCCCAACACTCATC |
| *GPR55* | ATCTACATGATCAACCTGGC | ATGAAGCAGATGGTGAAGACGC |
| *TRPV1* | TCACCTACATCCTCCTGCTC | AAGTTCTTCCAGTGTCTGCC |
| *PPARα* | TGGGAAGGCAGCGTTGATTA | CTGTGTCCTTCCCACTCTCG |
| *PPARγ* | TGATGTCTTGACTCATGGGTGT | CACGGAGCTGATCCCAAAGT |
| *PPARδ* | AGGTTCCCCAAGAGGGAAGA | CAGGAGGAGACAGTTCCAACC |
| *NAPE-PLD* | TTGTGAATCCGTGGCCAACATGG | TACTGCCATGGTGAAGCACG |
| *FAAH* | CCCAATGGCTTAAAGGACTG | ATGAACCGCAGACACAAC |
| *DAGLα* | AATGGCTATCATCTGGCTGAGC | TTCCGAGGGTGACATTCTTAGC |
| *DAGLβ* | GCGCAAAGTAAACGGCAAGA | CTGCAGCTTGGGCTTTTCAT |
| *MAGL* | ATGCAGAAAGACTACCCTGGGC | TTATTCCGAGAGAGCACGC |
| *ACTB* | TGACCCAGATCATGTTTGAG | TTAATGTCACGCACGATTTCC |
| *GAPDH* | CAGCCTCAAGATCATCAGCA | TGTGGTCATGAGTCCTTCCA |

| Antibody’s code | Brand | Diluition |
| --- | --- | --- |
| CB_1_ #101500 | Cayman Chemical (MI, USA) | 1:200 |
| CB_2_ #101550 | Cayman Chemical (MI, USA) | 1:200 |
| GPR55 #10224 | Cayman Chemical (MI, USA) | 1:200 |
| TRPV1 #TA336871 | OriGene (MD, USA) | 1:1000 |
| PPARα #SAB4502260 | Sigma-Aldrich (MO, USA) | 1:1000 |
| PPARγ #2443 | Cell Signaling Technology (MA, USA) | 1:1000 |
| PPARδ #PA1-823A | Invitrogen (MA, USA) | 1:750 |
| NAPE-PLD #10305 | Cayman Chemical (MI, USA) | 1:200 |
| FAAH #101600 | Cayman Chemical (MI, USA) | 1:200 |
| DAGLα #PA5-23765 | Invitrogen (MA, USA) | 1:1000 |
| DAGLβ #12574 | Cell Signaling Technology (MA, USA) | 1:1000 |
| MAGL #10212 | Cayman Chemical (MI, USA) | 1:200 |
| β-Actin #4970 | Cell Signaling Technology (MA, USA) | 1:1000 |

***Western Blotting***

**Table S3.** Primary antibodies used for Western Blotting analyses. All the primary antibodies used for ECS and for the housekeeping protein (β-Actin) are indicated.

***MAPK kinases expression in the inflamed model of keratinocytes***

**Figure S2.** MAPK kinases expression in the inflamed model of keratinocytes. **(A)** Representative images of phospho-kinase arrays for each treatment, captured by C-DiGit blot scanner. Each membrane detects the following 17 MAPKs: serine/threonine kinase 1 (AKT); cyclic adenosine monophosphate (cAMP) response element-binding protein (CREB); Glycogen synthase kinase 3 α (GSK3α) and -β (GSK3β); c-Jun N-terminal kinase (JNK); extracellular signal-regulated kinase (ERK1); mitogen-activated protein kinase (MEK1); Mitogen-activated protein kinase kinase 3 (MKK3) and 6 (MKK6); mitogen- and stress-activated protein kinase 2 (MSK2); Heat shock protein 27 (HSP27); mammalian target of rapamycin (mTor); p38 mitogen-activated protein kinase (p38); tumor protein p53 (p53); p70 ribosomal S6 kinase (P70S6k), Ribosomal S6 kinase 1 (RSK1) and 2 (RSK2) (each spotted in duplicate). The pairs of dots in the upper corner on the left side are positive controls. **(B)** Distribution of antibodies for each of the 17 MAPK phosphorylated proteins on the membrane supplied by the array.


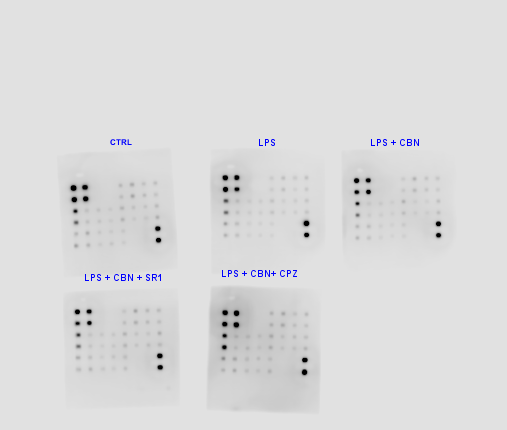


**(A)**


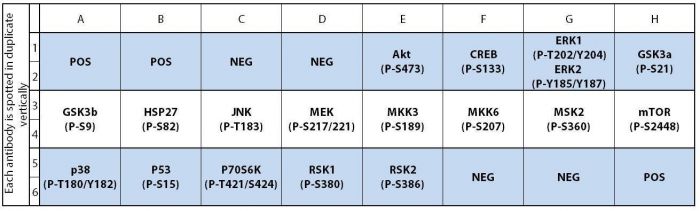


**(B)**
